# 

**Published:** 1984-01

**Authors:** 


Contents

3. Intra-thoracic Goitre with life threatening complications; a continuing diagnostic problem
E. Andrianopoulos and G. Keen

8. An unusual Pulmonary Embolus
John R. Fraser, M. Lansdown and Paul R. Godard

11. Total Parenteral Nutrition—A team for Central Venous Line Insertion at the Bristol Royal Infirmary; results of a Pilot Scheme
Arthur Allan, William Sellers, Neil Mortensen and David Leaper

14. Doctor Thomas Dimsdale, and Smallpox in Russia
John Griffiths

17. From our Foreign Correspondents

17. An Orthopaedic Surgeon in Malawi
A. L. Eyre-Brooke

21. Letter from America
Alastair Look

23. From our Correspondents

27. South West Urological Association

31. Association of Clinical Pathologists, Wessex Branch

34. Book Review

*continued overleaf*

Established 1883

### BRISTOL MEDICO-CHIRURGICAL JOURNAL

Incorporating the Medical Journal of the South West

34. Correspondence

35. Miscellanea

36. Examination Results

